# Blood lead, cadmium and mercury in relation to homocysteine and C-reactive protein in women of reproductive age: a panel study

**DOI:** 10.1186/s12940-017-0293-6

**Published:** 2017-08-08

**Authors:** Anna Z. Pollack, Sunni L. Mumford, Lindsey Sjaarda, Neil J. Perkins, Farah Malik, Jean Wactawski-Wende, Enrique F. Schisterman

**Affiliations:** 1 Department of Global and Community Health, College of Health and Human Services, George Mason University, 4400 University Drive MS5B7, Fairfax, VA 22030 USA; 2 Epidemiology Branch, Division of Intramural Population Health Research, Eunice Kennedy Shriver National Institute of Child Health and Human Development, National Institutes of Health, Bethesda, MD USA; 3 Department of Epidemiology and Environmental Health, School of Public Health and Health Professions, University at Buffalo, Buffalo, NY USA

**Keywords:** C-reactive protein, Cadmium, Homocysteine, Inflammation, Lead, Mercury, Women

## Abstract

**Background:**

To examine the relationship between cadmium, lead, and mercury concentrations with high-sensitivity C-reactive protein (hs-CRP) and homocysteine in women.

**Methods:**

Metals were measured at enrollment in whole blood. Homocysteine and hs-CRP were measured in one (*N* = 9) or two (*N* = 250) menstrual cycles up to 3 and 8 times per cycle, respectively. Linear mixed models with inverse probability of exposure weights to account for time varying confounding were used and models were stratified by dietary and serum vitamin status (dietary: vitamin B_6_, B_12_, folate; serum: folate).

**Results:**

Geometric mean (95% confidence interval (CI)) concentrations for cadmium, lead, and mercury were 0.29 (0.26–0.31) μg/L, 0.91 (0.86–0.96) μg/dL, and 1.05 (0.93–1.18) μg/L, respectively. Lead was associated with increased homocysteine (0.08; 95% CI: 0.01, 0.15) and this persisted among those in the lower three quartiles of consumption of vitamin B_6_, B_12_, folate, and serum folate but was not significant among those in the upper quartile. No associations were observed between metals and hs-CRP.

**Conclusions:**

Blood lead was associated with increased homocysteine in a cohort of healthy, premenopausal women but these associations did not persist among those consuming ≥75th percentile of essential micronutrients. Cadmium, lead, and mercury were not associated with hs-CRP concentrations.

## Background

A growing body of research points to associations between metals and the inflammation markers homocysteine and high sensitivity C-reactive protein (hs-CRP). Lead may increase homocysteine concentrations by interacting with thiols involved in one-carbon metabolism of homocysteine production [1]. Limited evidence suggests an association between cadmium, lead, and homocysteine in US adults [2], and between mercury and homocysteine in children [3]. Among older adults, populations with higher lead concentrations, and occupationally exposed workers, lead was associated with increased homocysteine concentrations [1, 4–6]. Cadmium was linked with hs-CRP in a study of cardiovascular events [7], in a cross-sectional study of US adults [8], and this association was attenuated among those with higher dietary antioxidant intake [9]. The blood lead-homocysteine association was stronger in older men with below-median micronutrient concentrations [10] and in a cross-sectional study of US adults [5]. Micronutrients play an important role in homocysteine/methionine metabolism, and B vitamins in particular can decrease homocysteine concentrations [11]. Although little is known about the modifying role that dietary micronutrient intake may have, these findings suggest that micronutrient levels may play an important role in the relationship between metals and homocysteine and hs-CRP.

However, research on homocysteine and hs-CRP in relation to metals concentrations in reproductive-aged women is limited. Cardiovascular disease (CVD) is the leading cause of death in U.S. women [12, 13] and modifiable factors offer important avenues for prevention. Total plasma homocysteine (tHcy) and hs-CRP are considered CVD risk biomarkers [14–16]. Both hs-CRP and homocysteine reflect distinct cellular processes that may contribute to inflammation and thus evaluating both biomarkers may provide greater insight as the specific mechanism by which metals influence CVD risk remains unknown.

Given limited research in reproductive aged women, our objective was to examine associations between cadmium, lead, and mercury with homocysteine and hs-CRP and to examine these associations stratified by methyl-donor nutrient dietary consumption (folate, vitamin B_6_ and B_12_) and serum folate concentrations in healthy, reproductive-aged women. Testing of this hypothesis was made possible by utilizing an existing high-quality data set.

## Methods

The BioCycle Study was a prospective cohort study that followed 259 healthy, premenopausal women aged 18–44 from Buffalo, New York, for up to two menstrual cycles. The majority of women participated in consecutive menstrual cycles. Of the 24 non-consecutive cycles, the median time interval between cycles was 30 days (IQR 27, 45), and the mean interval was 43.5 days. Recruitment occurred from 2005 to 2007. Details regarding the BioCycle Study have been published [17]. Briefly, participants provided self-reported demographics and information including reproductive history, smoking, and alcohol consumption. Participants were selected to be free of chronic disease and were not taking medications. Height and weight were measured using standard protocols by trained study staff to determine body mass index (BMI) (kg/m^2^). The University at Buffalo Health Sciences Institutional Review Board (IRB) approved the study and served as the IRB designated by the NIH for this study under a reliance agreement. All participants provided written informed consent.

### High sensitivity C-reactive protein and homocysteine measurement

Fasting blood was drawn in the morning during clinic visits, which were scheduled to correspond to approximately the second day of menstruation, mid- and late-follicular phase, 2 days around expected ovulation, and early, mid-, and late-luteal phase in each cycle. Within 90 min of the blood draw, all samples were processed and frozen at −80 °C and were shipped as complete individual cycle batches to the analytical laboratory. Ninety-four percent of study participants attended at least 7 of 8 scheduled clinic visits per cycle and 100% completed at least 5 clinic visits per cycle.

C-reactive protein was measured at each clinic visit in serum using high sensitivity methods on the IMMULITE 2000 (Siemens Medical Solutions Diagnostics, Deerfield, IL, USA), a chemiluminescent immunoassay sensitive to 0.3 mg/l [18]. Hs-CRP is considered a more sensitive assay for cardiovascular disease prediction compared with CRP according to Centers for Disease Control and Prevention and the American Heart Association guidelines [19, 20]. Homocysteine was measured 3 times each cycle (mid-follicular, ovulation, and mid-luteal menstrual cycle phase) in serum at the Kaleida Laboratory in Buffalo, New York, by chemiluminescence with Siemens IMMULITE 2000 homocysteine competitive immunoassay (coefficient of variation [CV] < 10.4%).

### Metals measurement

Whole blood was collected at study enrollment, which occurred approximately 2 weeks prior to the first cycle study visit. Samples were collected in purple-top tubes that were pre-screened to be free of trace metal contamination (Becton, Dickinson, and Company, Franklin Lakes, NJ). Centers for Disease Control (CDC) provided the collection tubes and protocols for collection, storage, and transport. Samples were refrigerated and sent to the Division of Laboratory Sciences, National Center for Environmental Health, for lead, cadmium, and mercury assessment by inductively coupled plasma mass spectrometry (ICP-MS). The interassay CV for cadmium, lead, and mercury were 4.3, 2.6 and 3.2%, respectively. Machine read values were used and values below the limit of detection (LOD) were not substituted to minimize bias [21, 22]. The LODs for cadmium, lead, and mercury were 0.20 μg/L (25% < LOD), 0.25 μg/dL (0% < LOD), and 0.30 μg/L (12% < LOD), respectively.

### Nutrient and hormone assessment

Nutrient intake of dietary folate, vitamin B_6_, B_12_, fish consumption, and omega-3 fatty acids, were assessed four times per cycle using a 24-h dietary recall questionnaire, for up to eight recalls total. Mean fish consumption was determined for each participant. As these components were measured by the same instrument, recall or other errors may be interdependent. Dietary intake data were collected and analyzed using the Nutrition Data System for Research software version 2005 developed by the Nutrition Coordinating Center, University of Minnesota. Eighty-seven percent of participants completed four dietary recalls per cycle and 99% completed 3 per cycle. Serum folate was measured at each clinic visit by the University of Minnesota from stored blood samples using a competitive protein binding assay and was performed with an Roche Elecsys 2010 Analyzer using the Roche Folate Gen 3 reagent (Roche Diagnostics, Indianapolis, Indiana) to detect 5-methyl tetrahydrofolate (THF), 5-formyl THF, and folic acid within the serum samples for each woman (CV <9.5%). Reproductive hormones were measured in fasting serum samples from all visits (5–8 visits/cycle) at the Kaleida Health Center for Laboratory Medicine (Buffalo, NY). Estradiol, progesterone, FSH, and LH were measured by solid-phase competitive chemiluminescent enzymatic immunoassay on the DPC Immulite 2000 analyzer (Siemens Medical Solutions Diagnostics) by Specialty Laboratories and CVs were <10% for estradiol, <5% for LH and FSH, and <14% for progesterone.

### Statistical methods

Descriptive statistics were evaluated for all covariates by homocysteine and hs-CRP dichotomized at their medians (hs-CRP: 0.8 mg/L; homocysteine: 5.9 μmol/L). Categorical variables were assessed using Pearson Chi-Square or Fisher exact tests while continuous variables were assessed using t-tests. Linear mixed models with log-transformed hs-CRP, homocysteine, and metals were run separately for each metal. Values greater than 10 mg/L of hs-CRP were excluded, as those values are indicative of current infection. A first-order autoregressive moving-average structure was specified for the correlation matrix. Random intercepts accounted for variation in hs-CRP and homocysteine concentrations between women and all measured values for hs-CRP and homocysteine were used. The mixed models accounted for the day of the menstrual cycle and cycle number for each woman. Inverse probability of exposure weighted models were implemented to account for time-varying confounding by micronutrient (Vitamin B_6_, B_12_, dietary and serum folate) and reproductive hormone concentrations (estradiol, progesterone, luteinizing hormone (LH), and follicle stimulating hormone (FSH)) [23, 24]. Confounders were selected based on a review of the literature and associations observed in descriptive analyses. Age, race (black, white, Asian, other), BMI, parity, smoking, and day of menstrual cycle were included in adjusted models. Results from continuous models are presented as a percent change in the outcome values per one-percent increase in the non-transformed exposure. To evaluate the potential for an interaction between metal exposure with homocysteine and hs-CRP by intake of B vitamins and folate or serum folate, models were stratified at the 75th percentile.

To examine the robustness of our findings, sensitivity analyses were run. First, a co-exposure model with all metals included together in the same model and a composite metal exposure category was created whereby if a participant was in the high category of exposure for any two metals, she was considered as high, whereas if a participant was in the low category for any two metals, she was considered low and otherwise was in the middle category of exposure. Tertiles were categorized as, cadmium: 0.04–0.22 μg/L, 0.23–0.36 μg/L, and 0.37–3.1 μg/L; lead: 0.31–0.72 μg/dL, 0.73–1.0 μg/dL, 1.1–6.2 μg/dL; mercury: 0–0.8 μg/L, 0.83–1.6 μg/L, 1.7–9.9 μg/L. A secondary analysis with fish consumption and omega-3 fatty acids were included as potential confounders both together and separately in the mercury exposure models. Statistical significance was defined as *p* < 0.05 and SAS version 9.4 was used for all analysis (SAS Institute, Cary, NC, USA).

## Results

Overall, women were young (mean 27.4, SD 8.2 years), of normal BMI (24.1, SD 3.9 kg/m^2^), did not smoke (96%), and were nulliparous (74%) (Table 1). Geometric mean concentrations of cadmium, lead, and mercury were 0.29 μg/L, 0.93 μg/dL, and 1.03 μg/L, respectively. Median concentrations of hs-CRP and homocysteine were 0.56 mg/L (range 0.10, 151.0) and 5.9 μmol/L (range 2.0, 12.9). No participants had levels that classified them as having high homocysteine (>15 μmol/L). One percent of hs-CRP values were >10 mg/L, indicative of current infection and 9% of values were >3 mg/L, the American Heart Association cutoff for elevated risk [25]. The majority of participants identified as white (57%). Those with hs-CRP concentrations above the median were older (29.5 vs. 25.4 years), had higher BMI (25.9 vs. 22.5 kg/m^2^), and were more often parous (36 vs. 17%) compared to women with lower hs-CRP concentrations. Those with homocysteine concentrations above the median had higher concentrations of lead, and lower serum folate, dietary folate, vitamin B_12_ (Table 1).

**Table 1:** Participant characteristics by high-sensitivity C-reactive protein (hs-CRP) and plasma homocysteine (tHcy) in women, Buffalo, NY

	Population (*n* = 259)	hs-CRP <0.56 mg/L^†^	hs-CRP ≥0.56 mg/L^†^	*p*-value^‡^	tHcy <5.9 μmol/L^†^	tHcy ≥5.9 μmol/L^†^	*p*-value^‡^
Age, years	27.4 ± 8.2	25.4 ± 7.3	29.5 ± 8.6	<0.01	28.1 ± 8.4	26.9 ± 8.1	0.28
BMI, kg/m^2^	24.1 ± 3.9	22.5 ± 2.8	25.9 ± 4.1	<0.01	23.9 ± 3.7	24.3 ± 4.0	0.36
Cadmium, μg/L^a^	0.29 (0.26, 0.31)	0.29 (0.26, 0.32)	0.28 (0.25, 0.32)	0.85	0.26 (0.23, 0.30)	0.30 (0.27, 0.34)	0.12
Lead, μg/dL^a^	0.91 (0.86, 0.96)	0.92 (0.85, 1.00)	0.90 (0.84, 0.98)	0.78	0.83 (0.77, 0.90)	0.97 (0.90, 1.05)	<0.01
Mercury, μg/L^a^	1.05 (0.93, 1.18)	1.03 (0.86, 1.22)	1.07 (0.90, 1.28)	0.72	1.14 (0.94, 1.37)	0.99 (0.84, 1.17)	0.28
Estradiol, pg/mL^a, b^	51.22 (48.60, 53.98)	50.2 (45.4, 55.5)	50.4 (44.4, 56.7)	0.95	52.6 (46.4, 59.6)	48.9 (44.3, 53.9)	0.35
FSH, mIU/mL^a, b^	6.59 (6.39, 6.79)	6.51 (6.16, 6.88)	6.50 (6.03, 7.00)	0.97	6.40 (5.92, 6.90)	6.58 (6.21, 6.96)	0.94
LH, ng/mL^a, c^	9.55 (8.87, 10.30)	9.35 (8.14, 10.74)	9.54 (8.14, 11.18)	0.85	9.39 (8.11, 10.88)	9.48 (8.18, 10.99)	0.93
Progesterone, ng/ml ^a, d^	6.16 (5.60, 6.77)	5.18 (4.18, 6.43)	6.41 (5.33, 7.71)	0.14	6.27 (5.20, 7.56)	5.22 (4.20, 6.49)	0.20
Serum folate, ng/ml^a^	19.89 (19.40, 20.38)	19.8 (18.9, 20.8)	20.0 (19.1, 21.0)	0.70	21.3 (20.2, 22.4)	19.1 (18.3, 19.9)	<0.01
Dietary folate, μg /day^a^	318.55 (303.0, 334.9)	331.4 (300.5, 365.5)	311.7 (281.0, 345.8)	0.40	349.2 (311.8, 391.2)	305.4 (278.9, 334.4)	<0.01
Dietary Vitamin B_6_, mg/day^a^	1.25 (1.19, 1.32)	1.32 (1.19, 1.45)	1.26 (1.13, 1.42)	0.60	1.38 (1.23, 1.54)	1.24 (1.12, 1.37)	0.17
Dietary Vitamin B_12_, μg /day^a^	2.53 (2.33, 2.76)	2.67 (2.24, 3.18)	2.64 (2.24, 3.10)	0.92	3.06 (2.16, 3.58)	2.42 (2.05, 2.86)	0.04
Alcohol, litre/day^a^	0.14 (010, 0.21)	0.13 (0.06, 0.27)	0.20 (0.09, 0.47)	0.43	0.14 (0.05, 0.38)	0.17 (0.09, 0.33)	0.76
Total calories, Kcal/day^a^	1482.8 (1433, 1534)	1454 (1355, 1560)	1487 (1378, 1605)	0.67	1562 (1432, 1702)	1415 (1328, 1508)	0.06
Race^e^							
White	148 (57)	71 (55)	77 (64)	0.18	56 (56)	92 (61)	0.24
Black	51 (20)	26 (20)	25 (21)		26 (26)	25 (17)	
Asian	35 (14)	24 (19)	11 (9)		11 (11)	24 (16)	
Other	25 (10)	8 (6)	7 (6)		7 (7)	8 (5)	
Smoking^e^							
No/former	249 (96)	125 (97)	114 (95)	0.53	98 (98)	141 (54)	0.30
Current	10 (4)	4 (3)	6 (5)		2 (2)	8 (5)	
Parity^e^							
0	187 (74)	109 (83)	78 (64)	<0.01	66 (65)	121 (80)	<0.01
≥ 1	66 (26)	22 (17)	44 (36)		36 (35)	30 (20)	

^a^Geometric mean (95% confidence limits) ^b^ Day 7 ^c^ Day 14 ^d^ Day 22

^e^Categorical variables [race, smoking, parity] expressed as n (%)

^†^tHcy and hs-CRP concentrations dichotomized on cycle 1 day 7

^‡^Continuous variables compared using t-test; categorical variables compared using Chi-square test or Fisher’s exact test

Each percent increase in lead concentrations was associated with a 0.08 μmol/L (95% CI: 0.01, 0.15) difference in homocysteine concentrations after adjustment for relevant confounding factors and inverse probability of exposure weights to account for time-varying confounding factors (Table 2). In unadjusted models, cadmium was associated with increased homocysteine 0.04 μmol/L (95% CI: 0.001, 0.09) but this association was not robust to adjustment for relevant confounding factors. Lead, cadmium, and mercury were not associated with hs-CRP.

**Table 2:** Cadmium, lead, and mercury association with homocysteine and c-reactive protein overall and stratified by dietary and serum micronutrients at the 75th percentile

	hs-CRP (mg/L)^a^ β (95% CI)		tHcy (μmol/L)^a^ β (95% CI)	
	Unadjusted	Adjusted ^b^	Unadjusted	Adjusted^b^
Cadmium^a^, μg /l	−0.03 (−0.22, 0.15)	−0.08 (−0.25, 0.08)	0.04 (0.001, 0.09)	0.03 (−0.01, 0.08)
Lead^a^, μg/dl	−0.05 (−0.31, 0.21)	−0.05 (−0.28, 0.19)	0.10 (0.04, 0.16)	0.08 (0.01, 0.15)
Mercury^a^, μg /l	−0.02 (−0.14, 0.11)	0.02 (−0.09, 0.12)	−0.01 (−0.04, 0.01)	−0.03 (−0.05, 0.003)
	Dietary Vitamin B_6_			
	<1.84 mg/day^b^	≥1.84 mg/day^b^	<1.84 mg/day^b^	≥1.84 mg/day^b^
Cadmium^a^, μg /l	−0.05 (−0.22, 0.12)	0.13 (−0.38, 0.12)	0.03 (−0.02, 0.08)	0.03 (−0.04, 0.09)
Lead^a^, μg/dl	−0.04 (−0.28, 0.20)	−0.02 (−0.37. 0.32)	0.07 (0.003, 0.14)	0.06 (−0.04, 0.15)
Mercury ^a^, μg /l	0.002 (−0.10, 0.11)	0.04 (−0.09, 0.19)	−0.02 (−0.05, 0.004)	−0.03 (−0.07, 0.01)
	Dietary Vitamin B_12_			
	<4.47 μg /day^b^	≥4.47 μg /day^b^	<4.47 μg /day^b^	≥4.47 μg /day^b^
Cadmium^a^, μg /l	−0.08 (−0.26, 0.09)	−0.17 (−0.40, 0.05)	0.02 (−0.02, 0.07)	0.06 (−0.01, 0.12)
Lead^a^, μg/dl	−0.02 (−0.27, 0.22)	−0.17 (−0.50, 0.16)	0.08 (0.01, 0.14)	0.05 (−0.03, 0.14)
Mercury^a^, μg /l	0.03 (−0.08, 0.13)	−0.003 (−0.15, 0.15)	−0.03 (−0.06, −0.005)	−0.01 (−0.05, 0.03)
	Dietary Folate			
	<456 μg /day^b^	≥456 μg /day^b^	<456 μg /day^b^	≥456 μg /day^b^
Cadmium^a^, μg /l	−0.09 (−0.27, 0.09)	−0.16 (−0.36, 0.04)	0.02 (−0.03, 0.07)	0.03 (−0.03, 0.10)
Lead^a^, μg/dl	−0.01 (−0.27, 0.24)	0.06 (−0.24, 0.36)	0.07 (0.0004, 0.14)	0.06 (−0.03, 0.15)
Mercury^a^, μg /l	−0.001 (−0.11, 0.11)	0.07 (−0.05, 0.20)	−0.03 (−0.06, 0.0001)	−0.03 (−0.07, 0.01)
	Serum Folate			
	<24.49 ng/ml^b^	≥24.49 ng/ml^b^	<24.49 ng/ml^b^	≥24.49 ng/ml^b^
Cadmium^a^, μg /l	−0.05 (−0.23, 0.13)	−0.24 (−.52, 0.04)	0.03 (−0.02, 0.08)	0.09 (0.02, 0.15)
Lead^a^, μg/dl	−0.07 (−0.32, 0.18)	0.01 (−0.39, 0.42)	0.07 (0.0001, 0.14)	−0.002 (−0.10, 0.10)
Mercury^a^, μg /l	0.03 (−0.08, 0.13)	0.03 (−0.14, 0.21)	−0.03 (−0.06, −0.005)	−0.01 (−0.05, 0.03)

*Abbreviations*: *hs-CRP* high sensitivity C-reactive protein, *tHcy* total serum homocysteine

^a^Natural log-transformed

^b^Adjusted for age, race (black, white, other), BMI, parity, smoking, and day of menstrual cycle with inverse probability of exposure weights that included time-varying confounding: vitamins (B_6_, B_12_, folate), reproductive hormone concentrations, calorie and alcohol intake

Potential modification of the associations between blood metals concentrations with hs-CRP and homocysteine by levels of dietary vitamins B_6_, B_12_, and folate, and serum folate were examined by dichotomizing vitamin B and folate at the 75th percentile and examining stratified associations. Lead was consistently associated with an increase in homocysteine concentrations among those with micronutrient levels below the 75th percentile (Table 2). Notably, among those with micronutrient levels ≥75th percentile, lead was not significantly associated with homocysteine (Table 2). Mercury was associated with decreased concentrations of homocysteine among those with serum folate <24.49 ng/ml, −0.03 μmol/L (95% CI: −0.06, −0.005) (Table 2). Micronutrient levels did not modify the association between metals and hs-CRP.

When simultaneously adjusting for metal co-exposure, each percent increase in lead remained associated with increased homocysteine, after adjustment for relevant confounding factors 0.10 μmol/L (95% CI: 0.03, 0.16) (Table 3). Cadmium and mercury were not associated with homocysteine or hs-CRP in the co-exposure models. Composite metals categories were not associated with changes in either hs-CRP or homocysteine concentrations (Table 3). A secondary analysis of mercury and homocysteine by additionally adjusting for mean fish consumption and omega-3 fatty acid levels both together and separately, which showed that the findings were robust to these additional potential confounders.

**Table 3:** Metal co-exposure in relation to high sensitivity-C reactive protein and total plasma homocysteine

	hs-CRP^b^ (mg/L)		tHcy^b^ (μmol/L)	
	Unadjusted	Adjusted ^a^	Unadjusted	Adjusted ^a^
Cadmium^b^, μg /l	0.18 (−0.16, 0.51)	0.17 (−0.20, 0.55)	0.03 (−0.02, 0.07)	0.02 (−0.03, 0.07)
Lead^b^, μg/dl	0.25 (−0.23, 0.73)	0.16 (−0.34, 0.66)	0.09 (0.03, 0.15)	0.10 (0.03, 0.16)
Mercury^b^, μg /l	0.08 (−0.13, 0.30)	0.09 (−0.13, 0.31)	−0.02 (−0.05, 0.01)	−0.02 (−0.05, 0.01)
Composite^c^				
Low	Ref	Ref	Ref	Ref
Medium	0.26 (−0.12, 0.63)	0.37 (−0.01, 0.74)	0.02 (−0.04, 0.08)	0.01 (−0.04, 0.07)
High	0.18 (−0.23, 0.59)	0.19 (−0.21, 0.60)	0.05 (−0.01, 0.12)	0.05 (−0.02, 0.11)

*Abbreviations*: *hs-CRP* high sensitivity C-reactive protein, *tHcy* total serum homocysteine

^a^Adjusted for age, BMI, smoking, race, average calories, cycle day and parity

^b^Natural log-transformed

^c^Composite tertiles definitions: high (in highest tertile of at least two metals), low (in lowest tertile of at least two metals), medium (all others)

## Discussion

To our knowledge, this is among the first studies to demonstrate that blood lead was associated with increases in homocysteine concentrations among healthy women with blood lead concentrations similar to or lower than the US population. In particular, the association between lead and homocysteine concentrations persisted among women consuming lower levels of essential B vitamins and folate. Specifically, geometric mean blood cadmium (0.29 μg/l [26] vs. 0.29 μg/l in the present study), lead (1.78 μg/dl [27] vs. 0.91 μg/l), and mercury (1.02 μg/l [28] vs. 1.04 μg/l) concentrations are similar to or lower than those observed among reproductive-aged women in the US. These findings suggest that even among young, healthy women, with relatively low exposure, higher lead concentrations are associated with higher homocysteine concentrations. This association remained after adjustment using weighted models to account for time varying confounding by micronutrient intake and reproductive hormone concentrations. Importantly, micronutrient intake modified the association and our findings suggest that lead was not associated with increased homocysteine concentrations for those with consumption of micronutrients ≥75th percentile.

Our finding that lead was associated with homocysteine is supported by several studies [1, 4, 6, 10, 29], which generally had higher lead exposures (average lead range: 3.5 to 22.7 μg/dL vs. 0.91 μg/dL in the present study) and older populations (mean age: 38–59 years vs. 27 in the present study) than the present study. A cross-sectional study among adults in Pakistan with average blood lead concentrations 11.7 μg/dL found greater changes in homocysteine 4.6% (95% CI: 2.6, 4.8%) per IQR increase (~8 μg/dL) in lead concentrations [6]. In contrast, lead exposures in our study were about 10 μg/dL lower. It is therefore notable that we observed similar findings in a healthy, younger population. A cross-sectional study of US adults, who were older than in the present study (mean age 50 vs. 27 years), found that the association between lead and homocysteine was modified by essential micronutrient concentrations [5]. Like Lee et al., who observed stronger associations between lead and homocysteine among those with lower concentrations of vitamin B_6_ and folate, here, the positive association between lead and homocysteine persisted among those with vitamin B_6_, B_12_, dietary and serum folate concentrations below the 75th percentile [5]. This is notable given differences by population age composition, sex, or concentration of homocysteine, as no one in the present study had concentrations that would be categorized as high (>15 μmol/L). A longitudinal study of older men (mean 69 years) with higher blood lead concentrations (4.9 vs. 0.91 μg/dl) similarly found that lead and homocysteine were more strongly associated among those with lower concentrations of folate and B vitamins [10], which was consistent with our findings of those below the 75th percentile of folate and B vitamins. Thus, our findings support the hypothesis that higher consumption of essential micronutrients may mitigate the observed association between lead and homocysteine among reproductive-aged women with low lead concentrations. This is striking given the health status of study participants and the relatively low concentrations of lead and homocysteine, along with fairly moderate levels of micronutrient intake. In particular, participants were selected who did not report special diets, which may explain the lower vitamin intake.

Mechanistically, lead may affect homocysteine concentrations via several pathways. Lead has been associated with oxidative stress, irregularities in the nitric oxide system, and inflammation [30, 31]. Lead can also interact with sulfhydryl groups, with homocysteine being a sulfur-containing amino acid [1, 32]. Lead exposure could lead to copper deficiency [33], and diets high in copper were associated with slight declines in folate and homocysteine concentrations [34], which may imply that diets low in copper could also affect homocysteine concentrations.

Mercury was associated with decreased homocysteine concentrations among those with serum folate <24.49 ng/ml. One study in children found a similar association among boys but not girls [3]. The mechanism of action by which mercury is associated with decreased concentrations of homocysteine in the presence of lower concentrations of serum folate may be by an increased need for glutathione, which causes homocysteine to be involved in glutathione production instead of methylating methionine [35]. This process would lead to lower homocysteine concentrations. Mercury has an affinity to selenium, an essential nutrient, which may play a role in its slightly negative association with homocysteine. Experimental evidence showed that plasma homocysteine was significantly reduced in selenium-deprived animals [36]. Additionally, diets rich in fish have been associated with reduced risk of coronary heart disease and fish consumption is a significant source of mercury exposure [37]. It is possible that the lower concentrations of homocysteine observed here were driven by fish intake and other lifestyle traits. While total fish consumption measured by 24-h dietary recall was low, median = 0 (range 0–12.2 servings), mean fish consumption was determined for each participant considering the up to eight 24-h dietary recalls completed in the study. Mean fish consumption was positively correlated with mercury concentrations (Spearman ρ = 0.42, *p* < 0.001) and mercury was weakly associated with omega-3 fatty acid levels (Spearman ρ = 0.06 *p* < 0.001). There are several possible reasons why metals were not associated with hs-CRP. A cross-sectional population-based study investigated these associations in an older population (age range 40–79 years) and found that urinary cadmium was associated with CRP [8]. Another cross-sectional study in an older population (mean female age 43.3 years) found urinary cadmium was significantly associated with increases in CRP, which were mitigated by adjustment for a composite antioxidant dietary score [9]. However, in the present study, the population was much younger, with a mean age of 27.4 years, and we did observe associations between age and hs-CRP. In this study, 95% of participants had concentrations of cadmium below 0.86 μg/l and median concentrations were 0.30 μg/l. Therefore, exposure concentrations may have been too low to observe such associations. The lack of association between CRP and metals may have been because the majority of the study population (61%) was of normal BMI, and CRP concentrations are tied to obesity-linked chronic inflammation [38]. BioCycle participants were selected to be free of chronic disease and medication use, and this selection strategy may have contributed to the null findings with respect to CRP.

This study had several limitations. Study generalizability may be limited to healthy, reproductive-aged women with fairly low concentrations of metal exposure. Further, this study had very low smoking prevalence, and thus findings may not be generalizable to populations with higher levels of smoking. The BMI of participants in the present study was lower than average, as most participants were in the normal range, which may limit generalizability. Other vitamins and trace elements such as zinc and copper are associated with inflammatory markers and could interact with metals but unfortunately were not measured in this study. Measurement error in metals, homocysteine, hs-CRP, or micronutrients may have biased our ability to observe associations. However, the fasting early-morning measurement of homocysteine may have mitigated some of the previously reported diurnal variability [39]. Should correlated measurement error have affected our findings, it is likely that this would have resulted in bias toward the null, which may mean that true associations were stronger than observed here [40].

However, this study had several strengths. Participants in the study were selected to be free from underlying chronic conditions, medication use, or special diets [17] to minimize unmeasured confounding. Multiple measurements of homocysteine and hs-CRP is another strength, as it has been shown that hs-CRP concentrations can change even over the menstrual cycle [41]. Further, consideration of modification by dietary and serum micronutrient levels among a population that did not consume special diets, medications, or supplements, is an additional strength. The consideration of time-varying confounding and the use of marginal structural models to appropriately account for such confounding minimizes the chance that observed associations were the result of confounding bias.

## Conclusions

We observed that lead was associated with increased homocysteine among healthy, young women. These findings suggest that even at low concentrations of lead exposure, among healthy women, lead may play a role in altering homocysteine concentrations, with potential implications for chronic diseases later in life. Among women with consumption of vitamins B_12_, B_6_, dietary and serum folate below the 75th percentile, lead was associated with higher concentrations of the inflammatory biomarker, homocysteine. Our findings showed that higher micronutrient concentrations may offer protection from the influence of lead on an important biomarker of cardiovascular risk.

## Abbreviations

BMI: Body mass index
CI: Confidence interval
CRP: C-reactive protein
CVD: Cardiovascular disease
hs-CRP: High-sensitivity-C-reactive protein
tHcy: Total plasma homocysteine
